# Three-dimensional imaging of upper tract urothelial carcinoma improves diagnostic yield and accuracy

**DOI:** 10.1172/jci.insight.175751

**Published:** 2024-07-22

**Authors:** Keishiro Fukumoto, Shigeaki Kanatani, Georg Jaremko, Zoe West, Yue Li, Kimiharu Takamatsu, Ibrahim Al Rayyes, Shuji Mikami, Naoya Niwa, Tomas Andri Axelsson, Nobuyuki Tanaka, Mototsugu Oya, Ayako Miyakawa, Marianne Brehmer, Per Uhlén

**Affiliations:** 1 Department of Medical Biochemistry and Biophysics, Karolinska Institutet, Stockholm, Sweden.; 2 Department of Urology, Keio University School of Medicine, Tokyo, Japan.; 3 Department of Oncology-Pathology, Karolinska Institutet, Stockholm, Sweden.; 4 Department of Diagnostic Pathology, National Hospital Organization Saitama Hospital, Saitama, Japan.; 5 Division of Urology, Danderyd Hospital, Stockholm, Sweden.; 6 Division of Urology, Department of Molecular Medicine and Surgery, Karolinska University Hospital, Stockholm, Sweden.; 7 Department of Urology and Department of Clinical Science and Education, Stockholm South General Hospital, Sweden.

**Keywords:** Oncology, Signal transduction, Urology

## Abstract

Upper tract urothelial carcinoma (UTUC) is a rare form of urothelial cancer with a high incidence of recurrence and a low survival rate. Almost two-thirds of UTUCs are invasive at the time of diagnosis; therefore, improving diagnostic methods is key to increasing survival rates. Histopathological analysis of UTUC is essential for diagnosis and typically requires endoscopy biopsy, tissue sectioning, and labeling. However, endoscopy biopsies are minute, and it is challenging to cut into thin sections for conventional histopathology; this complicates diagnosis. Here, we used volumetric 3-dimensional (3D) imaging to explore the inner landscape of clinical UTUC biopsies, without sectioning, revealing that 3D analysis of phosphorylated ribosomal protein S6 (pS6) could predict tumor grade and prognosis with improved accuracy. By visualizing the tumor vasculature, we discovered that pS6^+^ cells were localized near blood vessels at significantly higher levels in high-grade tumors than in low-grade tumors. Furthermore, the clustering of pS6^+^ cells was associated with shorter relapse-free survival. Our results demonstrate that 3D volume imaging of the structural niches of pS6 cells deep inside the UTUC samples improved diagnostic yield, grading, and prognosis prediction.

## Abstract 2

Examination of intact upper tract urothelial carcinoma biopsies with 3D imaging can predict tumor grade and prognosis more accurately than standard 2D imaging methods used in the clinic.

## Introduction

The incidence of upper tract urothelial carcinoma (UTUC) is relatively low, accounting for 5%–10% of urothelial cancers (1); however, at diagnosis, approximately 65%–70% of UTUCs are invasive, compared with 15%–25% of bladder cancers (2), and 7% are metastatic (3). Radical nephroureterectomy (RNU), which includes bladder cuff removal, is the gold-standard treatment for localized high-risk UTUCs (4). Despite the risk of recurrence (5), in recent years, kidney-sparing surgery (ureteroscopy with laser ablation in most cases) has been recommended in low-risk patients with UTUC. However, despite complete treatment, cancer can recur and progress in both the low- and high-risk groups and even cause patients to die from UTUC. Therefore, there is a need for tools to identify these patients at the time of initial diagnosis.

Efforts are being made to improve clinicians’ ability to evaluate and treat patients with UTUC. Sequenced DNA from formalin-fixed paraffin-embedded (FFPE) tumor specimens and urine sediments have demonstrated high diagnostic values for UTUC in recent studies (5, 6). Moreover, extensive work is underway to standardize guidelines pertaining to evaluation and treatment of patients with UTUC based on available evidence. Currently, the standard approach for diagnosing UTUC combines histopathological and cytological analyses (7, 8). The most widely used and accepted practices for grading urothelial tumors are the 1973/1999 and 2004/2016 World Health Organization systems (8, 9).

The diagnosis and grading of UTUC are heavily reliant on 2-dimensional (2D) imaging, which requires thinly sliced, stained, and mounted tumor samples. One complication of imaging UTUC samples is that they are often small (<1 mm^3^) and fragile. In addition, conventional histopathological methods use 2D microscopy, which cannot visualize 3D structures, such as the vasculature, resulting in the loss of crucial spatial information about the tumor environment (10). This can be resolved by utilizing DIPCO (diagnosing immunolabeled paraffin-embedded cleared organs), an imaging pipeline that captures images of intact tumor samples by 3D light-sheet microscopy (11). Efforts have been made using 3D imaging with increased sensitivity and accuracy for sentinel lymph node metastasis detection in melanoma patients (12).

Recently, markers of interest in urothelial carcinomas have targeted the PI3K/AKT/mTOR signaling pathway. Its activation has been shown to be correlated with reduced survival rates and increased tumor progression (13). Ribosomal S6 is a protein kinase and the endpoint of the PI3K/AKT/mTOR pathway, which regulates various cellular processes, including cell growth, proliferation, and apoptosis; therefore, it is an essential target for cancer treatment (14–16). Notably, increased expression of pS6 has been associated with poor prognosis in some cancers, including urothelial carcinoma (13, 17–19). Markers of vascularization are also of interest in cancer diagnostics because angiogenesis is required for tumor growth and metastasis, with increased expression of angiogenic markers linked to poor patient prognosis in some cancers (including urothelial bladder carcinoma) (20). Moreover, the vascularization of tumors differs from that of normal tissues in that the vessels are not smooth but have rough edges. Vessels are also narrower and prone to leakage, thus reducing blood flow; this results in a visually distinct vascularization pattern in tumors compared with normal tissues.

In this study, we show that 3D imaging of endoscopy biopsies can improve the diagnostic yield and accuracy of UTUC diagnoses. We developed a gelatin-embedding technique and 3D tissue-clearing imaging pipeline to overcome the clinical and pathological difficulties inherent in managing and diagnosing minute biopsies. Interrogating markers for the PI3K/AKT/mTOR pathway and the tumor vasculature, we show that 3D imaging can bridge the information gap that occurs when tumor specimens are studied with standard 2D microscopy and that advanced 3D analyzes can add value to conventional pathology, improving the accuracy of grade estimates.

## Results

### 3D imaging of intact UTUC biopsy samples.

**Figure 1:**
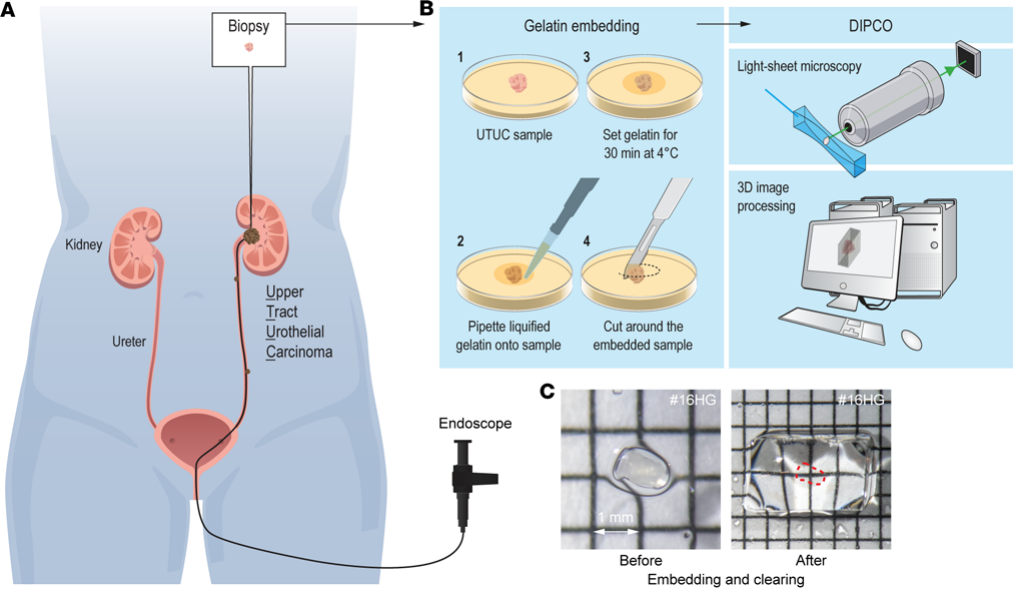
Pipeline for 3D imaging and gelatin embedding of UTUC samples. (**A** and **B**) Schematic representation of the endoscopic examination of patients with UTUC (**A**) and the DIPCO 3D light-sheet microscopy pipeline modified for gel embedding of small samples (**B**). (**C**) UTUC sample #16HG before and after gel embedding and clearing. Grid lines: 1 mm. The red dashed line indicates the cleared sample.

We sought to use state-of-the-art 3D imaging techniques to examine UTUC samples with the goal of improving pathological grading and prognosis. UTUC is located in the renal pelvis or ureter, and biopsies for pathological examination are taken during endoscopic procedures (Figure 1A). Since samples are minute and difficult to cut, we developed a method that could examine the tumor sample intact without having to section it (Figure 1B), based on our previously reported DIPCO pipeline that clears tumor samples and visualizes them using 3D imaging (11). To facilitate handling of the tiny (~1 mm) specimens, we embedded them in 3% gelatin before DIPCO (Figure 1C).

**Figure 2:**
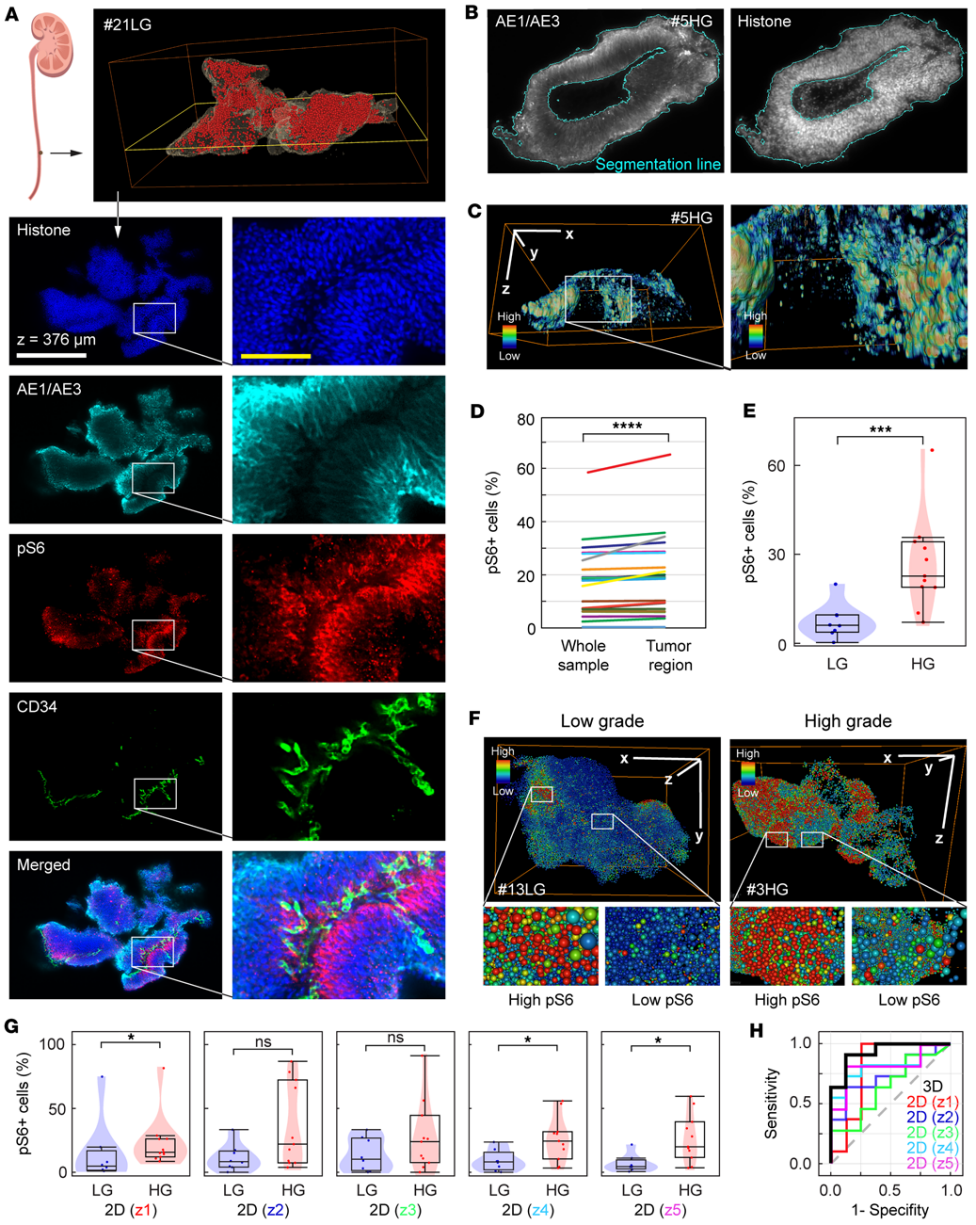
Whole-mount multiplex immunostaining of UTUC samples. (**A**) Schematic indicating where on the ureter UTUC sample #21LG was taken and which 2D layer showing the immunostaining for histone (blue), AE1/AE3 (cyan), pS6 (red), CD34 (green), and merged. (**B**) Cross section images of UTUC sample #5HG immunostained and segmented for AE1/AE3 and tumor region, respectively. Nuclei stained with histone nanobody. Segmentation lines in cyan. (**C**) Volume rendering of UTUC sample #5HG immunostained for pS6. Bounding box, 1,034 × 1,124 × 540 μm. (**D**) Cell-by-cell analysis of the percentage of pS6^+^ cells of the whole sample versus the tumor region (*n* = 21, ****P < 0.001 by Wilcoxon signed rank test). (**E**) Violin plot of the percentage of pS6^+^ cells in low-grade (*n* = 8) and high-grade (*n* = 11) UTUC samples (*P* = 0.002). (**F**) Volume rendering and cell-by-cell analysis of pS6^+^ cells in #13LG (left) and #3HG (right) UTUC samples. Bounding boxes, 786 × 540 × 470 μm (#13LG) and 1443 × 1304 × 644 μm (#3HG). (**G**) Violin plots of pS6^+^ cells in low-grade (*n* = 8) and high-grade (*n* = 11) UTUC samples analyzed for 5 randomly selected cross sections (z1-z5): z1, *P* = 0.026; z2, *P* = 0.083; z3, *P* = 0.302; z4, *P* = 0.021; and z5, *P* = 0.021.(**H**) ROC analysis of the pS6^+^ cell ratio in five 2D data sets (red, blue, green, magenta, and cyan) and the 3D data set (black). Scale bars: 100 μm (yellow), 250 μm (white), and *x*, *y*, *z* indicators 500 μm (white). The pseudocolors indicate low (blue) and high (red) expression levels of pS6. For the violin and box plots, the violin illustrates the distribution, the box center line indicates the median, the upper and lower boundaries of the indicate the upper and lower quartiles, and the whiskers indicate the minimum and maximum values. *P < 0.05, ***P < 0.005, ****P < 0.001 by Mann-Whitney *U* tests unless stated otherwise.

We next applied our modified DIPCO method to intact UTUC clinical samples, performing multiplex immunolabeling for histone, AE1/AE3, pS6, and CD34. We previously reported that histone nanobodies efficiently label nuclei in large 3D tissue volumes, enabling the counting of cells and determining their spatial location in a sample (21). The anticytokeratin antibody mixture AE1/AE3 recognizes acidic and basic epidermal keratins, with immunoreactivity in epithelial and most carcinoma cells. In this study, we labeled AE1/AE3 to outline the stromal parts in tumor specimens. The pS6 antibody was used to stain the phosphorylated form of the S6 protein, which has been associated with cancer progression, proliferation, and survival (22–25). To study the vasculature in UTUC, we assessed both CD31 and CD34 and concluded that CD34 showed more distinct vascular staining in our tissue volumes (Supplemental Figure 1; supplemental material available online with this article; https://doi.org/10.1172/jci.insight.175751DS1). The tumor vasculature is essential for tumor growth (26, 27), and an unorganized heterogeneous vasculature has been reported to associate with malignancy (11). DIPCO readily detected single nuclei labeled with histone and tumor cells of epithelial origin labeled with AE1/AE3 in intact UTUC samples (Figure 2A). Interestingly, when examining pS6 staining, a heterogeneous expression pattern was observed, with spatial niches of low- and high-density pS6^+^ cells spread throughout the samples. By staining for CD34, we detected a characteristic vasculature pattern throughout the UTUC sample (Supplemental Video 1).

### Spatial distribution analysis of pS6^+^ cells in UTUC.

To further investigate the inner landscape of UTUC samples, we used a cohort of 21 UTUC samples and performed a spatial cell-by-cell assessment (Supplemental Figure 2). Nuclear staining analysis of these 21 samples revealed that the number of cells ranged from 26,131 to 296,697. Labeling the tumor cells using AE1/AE3, DIPCO readily identified the tumor region within the intact UTUC sample (Figure 2B). Using 3D volume rendering and pseudo-coloring, we assessed the expression level and spatial location of pS6^+^ cells in 3D (Figure 2C). By overlaying the AE1/AE3^+^ tumor region with pS6 and histones, we performed cell-by-cell analysis to assess the expression level of pS6 in tumor cells at cellular resolution. Significantly more pS6^+^ cells were found in the tumor region than in the nontumor region (AE1/AE3^–^ cells) (Figure 2D; *P* < 0.001). Moreover, within the tumor region, the percentage of pS6^+^ cells was higher in high-grade UTUC than in low-grade UTUC (Figure 2E; *P* = 0.002). Further analysis, using pseudo-colored cell centroids to represent the level of pS6 expression for each cell, revealed that samples with overall low expression of pS6 had regions with high pS6 expression, and vice versa for samples with overall high pS6 expression (Figure 2F). Collectively, the results revealed substantial intratumoral heterogeneity (ITH) in the UTUC samples, encompassing both molecular and spatial ITH, which makes it more challenging to establish a diagnosis using 2D sections.

We then performed a comparative study between 3D and 2D image analyses used in daily clinical practice. Five 2D images (z1-z5) were randomly selected from each UTUC *Z*-stack by generating uniform random numbers. Two of the five 2D data sets failed to discriminate between the percentage of pS6^+^ cells in high- and low-grade tumors (Figure 2G; z1, *P* = 0.026; z2, *P* = 0.083; z3, *P* = 0.302; z4, *P* = 0.021; z5, *P* = 0.021), whereas this discrimination was notably more pronounced in 3D (Figure 2E; *P* = 0.002). To assess sensitivity and specificity, we plotted the percentage of pS6^+^ cells for 3D and 2D image analyses in a receiver operating characteristic (ROC) graph (Figure 2H). Calculation of the area under the curve (AUC) revealed that 3D analysis provided the highest accuracy in stratifying high- and low-grade UTUC (Table 1).

### Cluster analysis of pS6^+^ cells in UTUC.

**Figure 3:**
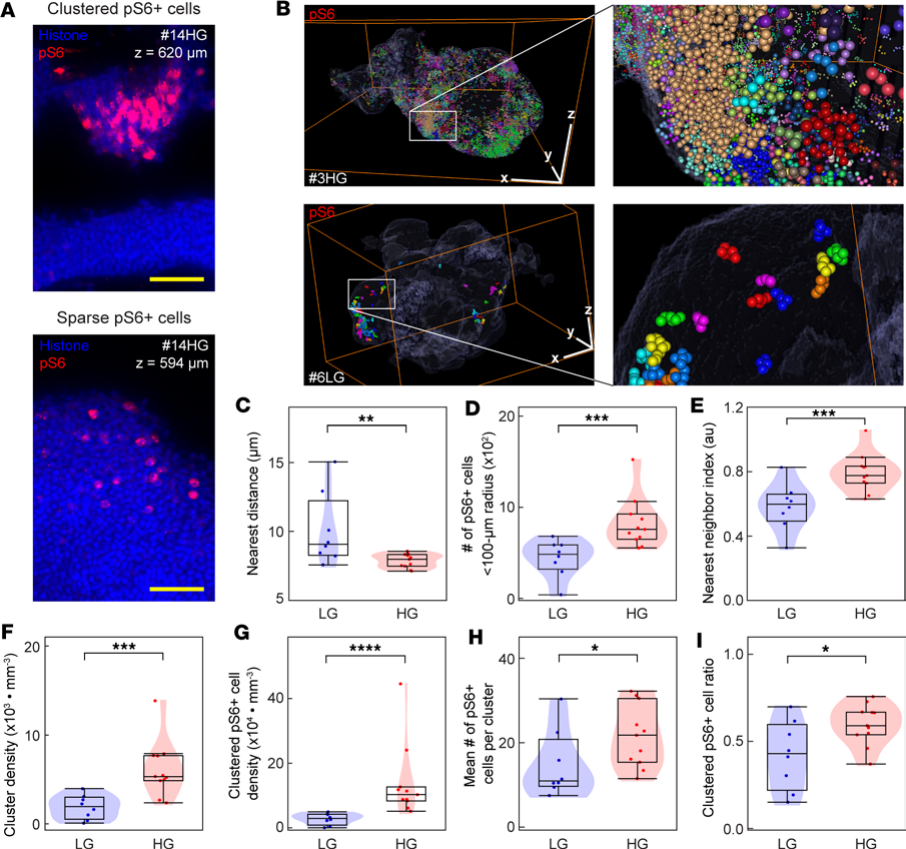
Cluster analysis of pS6^+^ cells in UTUC samples. (**A**) Cross sections of UTUC sample #14HG with clustered pS6^+^ cells at depth *z* = 620 μm (upper) and sparse pS6^+^ cells at depth *z* = 594 μm (lower). Nuclei stained with histone. (**B**) Volume rendering and cell-by-cell analysis of pS6^+^ cells in UTUC samples #3HG (upper) and #6LG (lower). Bounding boxes, 1,548 × 1,500 × 708 μm (#3HG) and 1,600 × 1,260 × 1,100 μm (#6LG). (**C**–**I**) Violin plots of pS6^+^ cells in low-grade (LG, *n* = 8) and high-grade (HG, *n* = 11) UTUC samples analyzed for nearest distance (**C**, *P* = 0.008), number of cells within a 100 μm radius (**D**, *P* = 0.003), nearest neighbor index (**E**, *P* = 0.004), cluster density (**F**, *P* = 0.002), clustered pS6^+^ cell density (**G**, *P* < 0.001), cells per cluster (**H**, *P* = 0.026), and clustered pS6^+^ cell ratio (**I**, *P* = 0.048). For the violin and box plots, the violin indicates the distribution, the box center line indicates the median, the upper and lower boundaries of the box indicate the upper and lower quartiles, and the whiskers indicate the minimum and maximum values. au, arbitrary unit. Scale bars: 50 μm (yellow), and *x*, *y*, *z* indicators 250 μm (white). *P < 0.05, **P < 0.01, ***P < 0.005, ****P < 0.001 by Mann-Whitney *U* tests.

When studying the spatial expression pattern of pS6 in our UTUC cohort, we observed an ITH pattern, with discrete regions of pS6^+^ cells that either formed clusters or were sparsely distributed (Figure 3A). A cluster analysis, where we stained the centroids of nearby cells with the same color, revealed that high-grade UTUC had more and larger clusters compared with low-grade UTUC (Figure 3B). To further characterize and quantify clusters of pS6^+^ cells, we explored various spatial parameters related to cell distribution and their association with tumor grade. The correlation between the mean nearest distance to a neighboring pS6^+^ cell and the tumor grade was examined. The nearest distance was shorter in high-grade tumors than in low-grade tumors, indicating that pS6^+^ cells were more prone to cluster in high-grade UTUC (Figure 3C; *P* = 0.008). Next, we counted the number of pS6^+^ cells within a sphere of a 100 μm radius from each pS6^+^ cell; the mean number of pS6^+^ cells within a 100 μm radius was significantly higher in high-grade tumors than in low-grade tumors (Figure 3D; *P* = 0.003). The nearest neighbor index (NNI) describes the spatial distribution and indicates whether a cell distribution pattern is clustered (NNI < 1) or dispersed (NNI > 1) (21). Calculating the NNIs for our UTUC samples showed that both low-grade and high-grade UTUC had NNI values that were < 1, indicating clustered cell patterns; however, the NNI value was significantly higher in high-grade than in low-grade tumors (Figure 3E; *P* = 0.004). The total number of clusters and pS6^+^ cells in each cluster in the low- and high-grade tumors were also assessed. Cluster density (Figure 3F; *P* = 0.002), clustered pS6^+^ cell density (Figure 3G; *P* < 0.001), the mean number of pS6^+^ cells per cluster (Figure 3H; *P* = 0.026), and clustered pS6^+^ cell ratio (Figure 3I; *P* = 0.048) were higher in high-grade tumors than in low-grade tumors. Together, these data show marked differences in the volumetric 3D imaging of pS6^+^ cells between low- and high-grade UTUC tumors.

### Analysis of the tumor vasculature and pS6 in UTUC.

**Figure 4:**
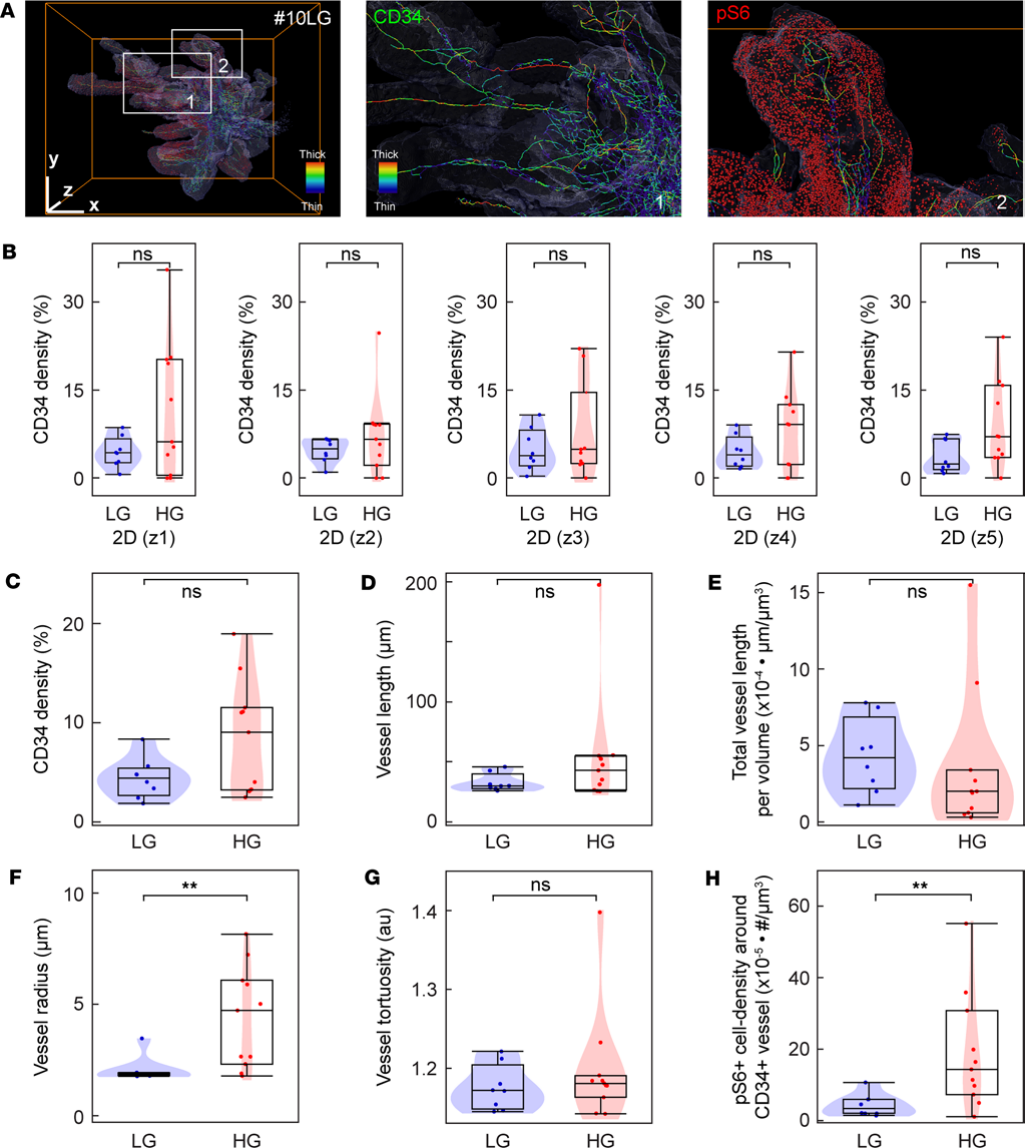
Assessment of the tumor vasculature and the density of pS6^+^ cells around vessels. (**A**) Volume rendering of the tumor vasculature and pS6^+^ cells in low-grade UTUC sample #10LG. The tumor vasculature was stained for CD34. The pseudocolors indicate thin (blue) and thick (red) blood vessels. Bounding box, 1,879 × 1,447 × 920 μm. (**B**–**H**) Violin plots of pS6^+^ cells in low-grade (LG, *n* = 8) and high-grade (HG, *n* = 11) UTUC samples analyzed for CD34 density in 5 randomly selected 2D data sets (z1–z5) (**B**, z1, *P* = 0.36; z2, *P* = 0.32; z3, *P* = 0.46; z4, *P* = 0.41; and z5, *P* = 0.069), 3D CD34 density (**C**, *P* = 0.22), mean vessel length (**D**, *P* = 0.22), total vessel length per volume (**E**, *P* = 0.14), vessel radius (**F**, *P* = 0.008), vessel tortuosity (**G**, *P* = 0.46), and pS6^+^ cell density around CD34^+^ vessel (**H**, *P* = 0.01). AU, arbitrary unit. For the violin and box plots, the violin indicates the distribution, the box center line indicates the median, the upper and lower boundaries of the box indicate the upper and lower quartiles, and the whiskers indicate the minimum and maximum values. **P < 0.01 by Mann-Whitney *U* test.

Tumor angiogenesis and vascular heterogeneity are associated with disease progression and malignancy (28). To explore the spatial relationship between pS6^+^ tumor cells and blood vessels, we sought to immunolabel UTUC tumors for pS6 alongside CD34 to stain vascular endothelial cells (Figure 4A). A reconstruction of the vasculature in 3D allowed us to analyze the CD34 density, vessel length, total vessel length per volume, vessel radius, and vessel tortuosity for each tumor. Since the CD34 density was the only parameter that could be assessed in 2D, we analyzed the CD34 density in five randomly selected 2D image sets and its relationship with low- and high-grade tumors. This analysis produced a clear result, as all 2D images failed to stratify between low- and high-grade tumors (Figure 4B). When assessing parameters in 3D, such as the CD34 density (Figure 4C), vessel length (Figure 4D), total vessel length per volume (Figure 4E), vessel radius (Figure 4F), and vessel tortuosity (Figure 4G), only the vessel radius showed a significantly higher radius in high-grade tumors compared with low-grade tumors (*P* = 0.008). Finally, we analyzed the number of pS6^+^ cells surrounding the blood vessels; notably, the density of pS6^+^ cells around the blood vessels was significantly higher in high-grade tumors than in low-grade tumors (Figure 4H; *P* = 0.01). Collectively, the results indicate that the blood vessel radius and density of pS6^+^ cells surrounding the blood vessels are markers that can stratify low-grade and high-grade UTUC.

### Clinical implications of 3D image analysis of UTUC.

**Figure 5:**
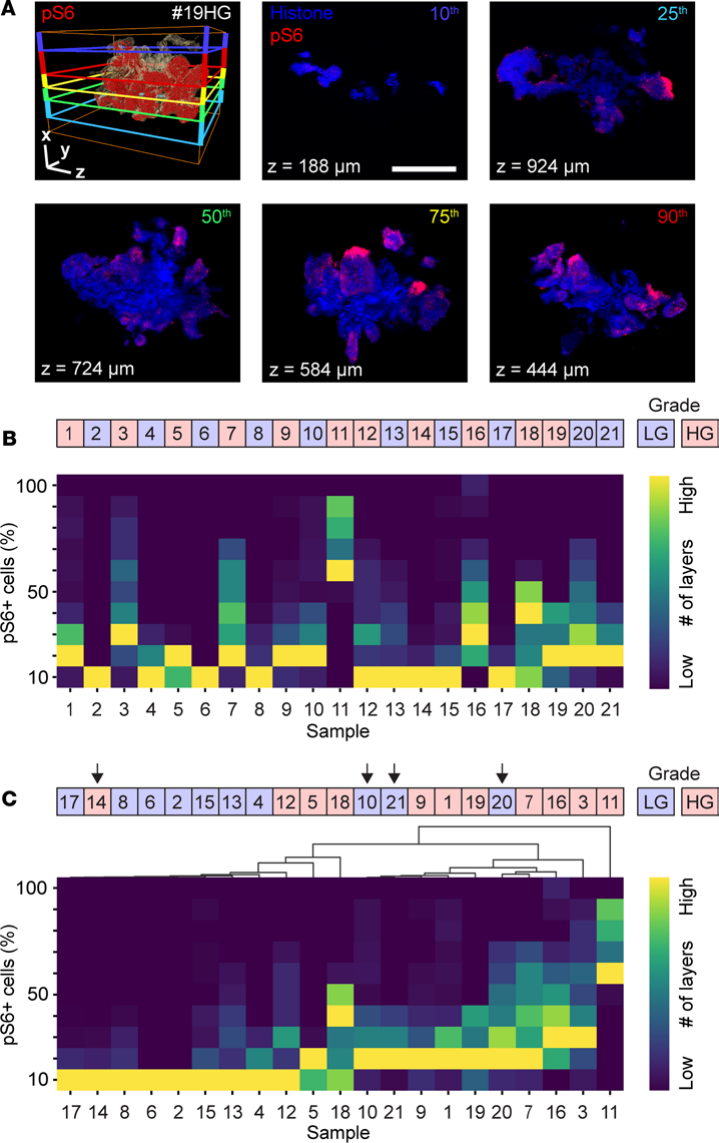
Analysis of intratumoral heterogeneity in UTUC samples. (**A**) A 3D rendering of UTUC sample #19HG and the 2D layers at the 10th (*z* = 188 μm, blue), 25th (*z* = 924 μm, cyan), 50th (*z* = 724 μm, green), 75th (*z* = 584 μm, yellow), and 90th (*z* = 444 μm, red) percentiles. Bounding box, 1,600 × 1,396 × 1,100 μm. Nuclei stained with histone. (**B** and **C**) Heatmap of all UTUC samples and their number of layers with the indicated percentage of pS6^+^ cells (**B**) and the corresponding hierarchical cluster analysis (**C**). High and low numbers of layers are indicated by yellow and dark blue, respectively. Red and blue boxes indicate high-grade (HG) and low-grade (LG) samples, respectively, diagnosed by the pathologist. Arrows indicate mismatched samples.

To assess the ability of intact UTUC 3D analysis to aid diagnostic and prognostic work in the clinic, we investigated whether pS6, cluster, and blood vessel analyses could detect erroneously graded samples and predict relapse. We first investigated the effect of ITH on histopathological diagnosis by studying five 2D layers in sample #19HG. To ensure these layers comprehensively represented the entire tumor, we selected them at equidistant points along the percentile scale: the 10th, 25th, 50th, 75th, and 90th percentiles. A clear pattern of ITH was observed, characterized by discrete regions showing high and low pS6 expression (Figure 5A). To further analyze the expression pattern of pS6^+^ cells in all layers and assess whether there were clear discrepancies between low- and high-grade tumors, we plotted a heatmap for the entire cohort (Figure 5B). Then we used hierarchical clustering analysis to group similar samples together. This analysis efficiently grouped low-grade and high-grade tumors into 2 clusters (Figure 5C), except for 4 mismatched samples, #14HG, #10LG, #21LG, and #20LG (marked with arrows). Samples #21LG and #20LG were biopsies taken from the same patient as #19HG but at different time points. Sample #20LG was graded as low-grade at the earliest time point; approximately 3 months later, #19HG was graded as high-grade, and 3 months after that, #21LG was graded as low-grade Notably, our 3D histopathological analysis clustered all 3 of these biopsies as high grade. This patient experienced multiple recurrences, suggesting high-grade UTUC and also suggesting that 2 of 3 samples had been erroneously graded. Sample #14HG was clinically diagnosed as high grade in the biopsy specimen; however, samples from subsequent nephroureterectomy were diagnosed as low grade, similar to the diagnosis of the 3D analysis. The patient has been recurrence free for 3 years after sample collection. Sample #10LG was pathologically diagnosed as a low-grade tumor; however, it was clustered with high-grade tumors in our 3D analysis.

**Figure 6:**
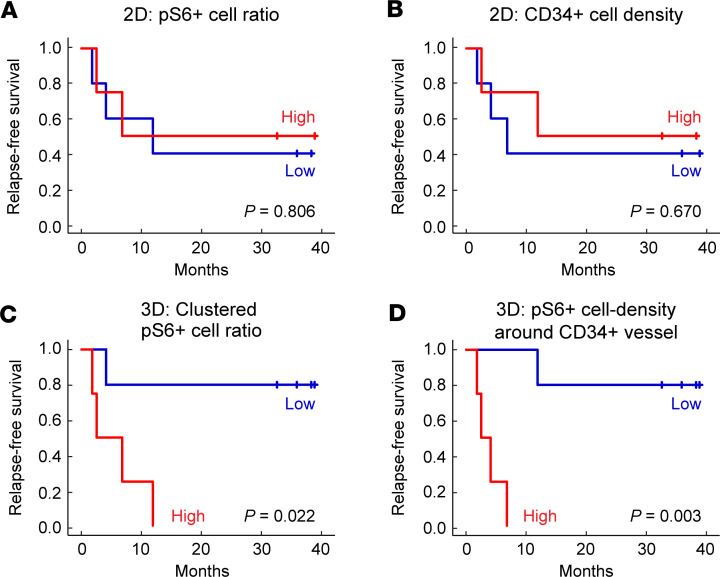
Relapse-free survival analysis in patients with UTUC who underwent focal therapy. Kaplan-Meier plots of relapse-free survival in patients with UTUC (*n* = 9) who underwent focal therapy. (**A**–**D**) Stratification of UTUC, based on 2D-analysis, into high versus low pS6^+^ cell ratio (**A**, *P* = 0.806) and CD34^+^ cell density (**B**, *P* = 0.670), and, based on 3D-analysis, into high versus low clustered pS6^+^ cell ratio (**C**, *P* = 0.022) and pS6^+^ cell density around CD34^+^ vessel (**D**, *P* = 0.003). Statistical analysis was performed using log–rank tests.

To evaluate the potential of the 3D imaging analysis to predict relapse-free survival, we performed Kaplan-Meier analyses on UTUC samples from patients who underwent focal therapy. We began by stratifying the cohort based on 2D imaging. Dividing the samples into groups of high versus low pS6^+^ cell ratio could not detect any difference in relapse-free survival (Figure 6A and Supplemental Figure 3A; *P* = 0.806). Moreover, a split into high versus low CD34^+^ cell density from 2D images failed to detect a difference in relapse-free survival (Figure 6B and Supplemental Figure 3B; *P* = 0.670). We next interrogated our 3D data and stratified the cohort into high versus low clustered pS6^+^ cell ratios. Patients with a low clustered pS6^+^ cell ratio exhibited significantly longer relapse-free survival (Figure 6C; *P* = 0.022). Additionally, we stratified the cohort based on the 3D imaging analysis of the density of pS6^+^ cells around the CD34^+^ vessels, revealing that the high-density group had significantly shorter relapse-free survival (Figure 6D; *P* = 0.003). These findings indicate that 3D analysis of the tumor microenvironment could serve as a tool to predict recurrence.

## Discussion

Here, we used 3D imaging to evaluate the expression of pS6, a downstream effector of the PI3K/AKT/mTOR pathway and a tumor marker (13, 14), in intact UTUC samples. Tumor tissues contain nonmalignant stromal and immune cells, which comprise the tumor environment and are closely related to tumor cells (29–32). We distinguished the tumor from the stromal region by immunolabeling with AE1/AE3. The percentage of pS6^+^ cells in the tumor was significantly associated with tumor grade, with high-grade tumors exhibiting a significantly higher percentage of pS6^+^ cells than low-grade tumors. The analysis of the percentage of pS6^+^ cells was more pronounced in 3D compared with the 2D analysis, suggesting that 3D analysis provides greater accuracy than 2D analysis. As 3D analysis provides an in-depth insight into the tumor environment, we obtained the spatial coordinates of all pS6^+^ cells and performed various spatial analyses (including cluster analyses). These results reveal significant associations with tumor grade; high-grade UTUC tumors demonstrated tighter clusters of pS6^+^ cells than low-grade UTUC samples. Additionally, there were more pS6^+^ cell clusters within high-grade UTUC tumors and more pS6^+^ cells within these clusters than in low-grade UTUC samples. This indicated that assessments of pS6^+^ cells may serve as a potential prognostic marker for UTUC diagnosis, as was also demonstrated by the Kaplan-Meier analysis of relapse-free survival in patients with UTUC who had undergone focal treatment. A correct diagnosis and prognosis is critical for patients with UTUC, as understaging may risk a patient’s life, while overstaging can subject the patient to unnecessary nephroureterectomy — i.e., removal of the kidney, the entire ureter, and a piece of the bladder — with the risk of losing kidney function and in some cases leading to hemodialysis.

Furthermore, we performed a structural analysis of blood vessels in intact UTUC samples. Evaluation of CD34 density did not show a significant correlation with tumor grade or relapse; however, analysis of the vascular structure based on CD34 staining revealed that the radius of the blood vessel correlated with tumor grade and relapse. High-grade UTUC had a significantly larger vessel radius, correlating with a lower relapse-free survival rate. Moreover, the pS6^+^ cell density around blood vessels was associated with both tumor grade and relapse; high-grade UTUC samples demonstrated a stronger association with high pS6^+^ cell density around the blood vessels and a lower relapse-free survival rate. Based on these data, the prognosis of UTUC can be estimated from an intact sample by analyzing the distribution of pS6^+^ cells rather than simply examining the degree of pS6 expression. The correlation between these spatial distributions and prognosis may be related to the cancer stem cell (CSC) niche; CSCs exhibit self-renewal, differentiation, plasticity, and therapeutic resistance (33). The PI3K/AKT/mTOR pathway is thought to play an important role in the carcinogenesis of CSCs, as it is required for their proliferation and colony formation (34–38). Therefore, our discovery of pS6^+^ cell clusters correlating with poor prognosis may be due to the cluster representing the CSC niche. Similarly, the positional relationship between pS6^+^ cells and blood vessels may be due to niche-stimulated angiogenesis (39, 40).

A 3D analysis of UTUC samples could provide a better perspective on tumor heterogeneity, which could be used for diagnosis, grading, and prognosis. The importance of tumor heterogeneity has been revealed by genome sequencing, which uncovered substantial heterogeneity among patients with similar cancer that arose during this evolutionary process (41, 42), demonstrating the need for personalized medicine (43, 44). Recent sequencing techniques, including single-cell RNA-Seq (scRNA-Seq), have deeply explored ITH and intertumoral heterogeneity in various carcinomas providing insight into carcinogenesis, CSCs, and the tumor microenvironment (including the immune system) at the single-cell resolution (30–32, 45–49). Since ITH plays a vital role in cancer progression and therapy failure (50, 51), an accurate understanding of ITH is indispensable for formulating an optimal treatment plan for patients. The traditional 2D microscope is often used for diagnosis in clinical practice; however, 2D imaging fails to analyze the 3D environment, leading to a lack of understanding of ITH. Furthermore, obtaining samples of sufficient size for proper analysis during surgery is not always possible.

We have previously reported that 3D imaging with DIPCO and DIIFCO is a powerful tool for evaluating whole clinical samples and ITH (11, 52). However, these protocols were optimally designed for samples that must be mounted on a light-sheet stand, which is approximately 5.50 × 3.33 mm; this becomes challenging with minute clinical specimens, such as UTUC, which are typically diagnosed using biopsies < 1 mm^3^. Therefore, we optimized the method of embedding minute clinical samples in gelatin to increase their mounting size to allow for intact tumor analysis. Appropriate staining and clearing results were obtained for all UTUC samples. However, this method is not limited to UTUC samples and can also be applied to other samples that are minute for standard light-sheet microscopy mounting methods. An advantage of mounting the samples in gelatin for microscopy is that the sample is easier to handle when it becomes transparent after clearing.

Our volumetric 3D imaging method and cell-by-cell analysis generated results that can aid pathologists in diagnosing UTUC more accurately. One of the advantages of this method is that it is possible to analyze all cells regardless of the sample size. The total number of cells in the samples ranged from 26,131 to 296,697, with 38% of the samples containing < 100,000 cells, making it challenging to perform scRNA-Seq on all cells. Additionally, 3D imaging allows analysis of the tumor with preserved structure, unlike scRNA-Seq where the sample is dissociated and the structure is destroyed. The lack of spatial information about the tumor’s architecture and microenvironment also applies to urine cytology and sequencing of urine sediment–derived DNA. Maintaining 3D structures provides a better analysis of ITH, as data can be collected on the spatial positional relationship between cells and the correlation with blood vessels and cell niches; these are lost with scRNA-Seq and 2D image analysis. Our results indicate that the spatial distribution analyses are superior to simple quantitative analyses of positive cells. Some key results, including cluster and vessel analyses, are illustrated, and findings that could not be detected with conventional 2D microscopy could be used to aid UTUC diagnosis, grading, and prognosis prediction. Another advantage of the 3D DIPCO imaging method is that it is possible to return FFPE samples to the biobank after analysis for future study (11). Presently, light-sheet microscopy is rare in hospital settings; however, the running cost of performing DIPCO is low, and the cost of a light-sheet microscope is within $200,000 to $400,000 (USD) (53). 3D image analysis is in its infancy, challenged with problems such as speed, cost, optimized sample preparation, and image-processing methods, which must be solved to complete the pipeline for 3D image analysis in clinical practice. Notably, many technological advances in light-sheet microscopy (10), tissue clearing (54), and machine learning (55, 56) are currently underway.

In the current clinical practice, diagnosis and treatment strategies are determined based on limited 2D information from biopsy and cytology samples. In this study, we revealed that 3D image analysis of minute biopsies identified key tumor features that could aid in UTUC diagnosis, grading, and prediction, which were distorted or lost in 2D image analysis. This suggests that 2D analysis of tumor samples may not accurately represent the tumor environment, leading to cases of understaging and overstaging. In the future, 3D analysis, including DIPCO sample preparation as used in this study, may become a tool for clinical pathologists when facing difficult samples that are challenging to stage or grade. By accumulating a larger number of UTUC cases and performing retrospective and prospective 3D imaging studies, it may be possible to improve the standard clinical grading schemes. This could allow for more accurate and personalized treatment strategies for patients with UTUC.

## Methods

### Sex as a biological variable.

The participants in our study were randomly selected, including both male and female patients with UTUC. Sex was not considered a biological variable.

### Sample collection and fixation.

Twenty-one clinical UTUC samples were collected from 19 patients between 2017 and 2020 using biopsy forceps (Piranha, Boston Scientific) at Danderyd Hospital. The tissues were fixed with 4% paraformaldehyde (Sigma-Aldrich) after surgery. Of the 21 clinical samples, 17 (#1–17) were stored in methanol (Sigma-Aldrich), and the remaining 4 (#18–21) were embedded in paraffin (Sigma-Aldrich). The samples were ciphered with numbers to avoid investigator bias during image and data analysis. All samples were histologically confirmed to have UC. These tumors were staged according to the 2002 TNM staging system approved by the Union for International Cancer Control (UICC). The histological grades were assigned according to the 1999 and 2004 World Health Organization grading systems. All histopathological examinations were performed by hospital pathologists.

### Sample preparation.

Paraffin-embedded tissue samples were deparaffinized by xylene (Sigma-Aldrich) treatment at 37°C for 1 hour; tissue samples were subsequently treated with xylene for 1 hour at room temperature (RT). The samples were washed twice with 100% ethanol (Histolab) for 1 hour and once with 100% methanol for 1 hour. Next, samples were bleached using 5% hydrogen peroxide (Sigma-Aldrich) in methanol at RT for 1 hour or 10% hydrogen peroxide in methanol at 55°C for 2 hours, according to the conditions of the samples. After bleaching, the samples were rehydrated with a series of methanol solutions of decreasing concentrations (80%, 60%, 40%, and 20%) for 15 minutes and washed once in PBS for 15 minutes and twice in PBS (Thermo Fisher Scientific) with 0.2% Triton X-100 (Sigma-Aldrich) (PTx.2) for 15 minutes.

### Whole-mount staining.

The methanol-treated samples were permeabilized in permeabilization buffer (0.2% Triton X-100, 20% DMSO [Sigma-Aldrich], and 0.3M glycine [Sigma-Aldrich] in PBS) at 37°C for 1 hour. Next, samples were blocked using blocking buffer (0.2% Triton X-100, 10% DMSO, and 6% donkey serum [Jackson ImmunoResearch] in PBS) at 37°C for 1 hour and incubated with primary antibodies in PBS with 0.2% Tween-20 (Sigma-Aldrich) and 10 μg mL^−1^ heparin (Sigma-Aldrich) (PTwH), 5% DMSO, and 3% donkey serum at 37°C for 2 days. The primary antibodies used were anti-CD34 (1:40, sheep polyclonal, AF7227; R&D Systems), anti–phospho-S6 Ribosomal Protein (1:100, rabbit monoclonal, 4858; Cell Signaling Technology), and anti–cytokeratin Pan Type I/II (AE1/AE3) (1:1,000, mouse monoclonal, MA1-82041; Thermo Fisher Scientific). The immunolabeled samples were washed in PTwH 3 times for 1 hour and incubated with secondary antibodies in PTwH with 3% donkey serum at 37°C for 2 days. The secondary antibodies (1:200) used were Alexa 555–conjugated donkey anti–sheep IgG H+L (ab150178, Abcam), Alexa 647–conjugated affinity-purified F(ab′) 2 fragment donkey anti–rabbit IgG H+L (711-606-152, Jackson ImmunoResearch Laboratories), and Alexa 790–conjugated affinity-purified donkey anti–mouse IgG H+L (715-655-150, Jackson ImmunoResearch Laboratories). ATTO 488–conjugated anti-histone nanobodies (1:500; tba488-100; ChromoTek) or YO-PRO-1 (1:5000; Y3603; Thermo Fisher Scientific) were added to the secondary antibody solution for nuclear staining. Finally, the samples were washed in PTwH for 1 day.

### Gel embedding and clearing.

The samples were embedded in gelatin (Sigma-Aldrich) by directly placing the immunolabeled UTUC biopsy in a 3% gelatin mold. Next, a drop of liquid gelatin (at 37°C) was added to the sample and allowed to cool to embed it in the gel completely. After cooling, the sample was cut to an appropriate size and cleared. For clearing, the gels were dehydrated using a series of methanol solutions of increasing concentration (20%, 40%, 60%, 80%, and 100%) for 1 hour each. After dehydration, the gel was treated with 66% dichloromethane (Sigma-Aldrich) in methanol for 3 hours and washed twice with 100% dichloromethane for 15 minutes. Finally, the gels were incubated overnight with dibenzyl ether (Sigma-Aldrich).

### Light-sheet microscopy.

Intact tumor samples were examined using a light sheet microscope (UltraMicroscope II, Miltenyi Biotec). The fluorescent probes were excited with laser lines at 488, 561, 639, and 785 nm, and 3D images were captured using a ×2 objective lens with a numerical aperture of 0.5 (MVPLAPO). The light sheet had a width of 3.75 μm (20% overlap), and images were recorded with an XY-resolution of 0.48 μm and a *Z*-step size of 2 μm. Raw images were collected as 16-bit TIF images and, if necessary, converted into an 8-bit TIF format for further analysis.

### Image stitching and registration.

Images acquired by light-sheet microscopy were stitched with ImageJ (NIH) (57) or TeraStitcher (58). After stitching, the position of each channel image was matched to the nuclear staining channel as a reference using the registration function of Amira (Thermo Fisher Scientific). To increase the accuracy, original images were preprocessed with the 639 nm channel images and the “Background Detection Correction” function in Amira.

### Single-cell analysis.

Single-cell analysis was performed by segmenting nuclei stained with histone or YO-PRO-1 by preprocessing images with ImageJ (“Unsharp Mask” and “Normalize Local Contrast” function) and Amira (“Gaussian Filter” and “Background Detection Correction” function) software. The H maxima algorithm in Amira was used to detect the intensity maxima corresponding to the cells’ center regions. The number of cells and the centroid position of each cell were obtained by analyzing connected components with MATLAB (Mathworks) (“bwconncomp” function). The expression of pS6 in individual cells was analyzed by calculating the mean signal intensities in a circular ROI (17 pixels, 8.16 μm diameter) aligned with the centroid of each cell using custom MATLAB scripts. The 10th, 25th, 50th, 75th, and 90th percentiles were derived with Excel (Microsoft) (“percentile.exc” function), and layers below the 10th percentile were considered the tumor’s edge and excluded from the analysis.

### Cluster analysis.

For the cluster analysis, the distance between all cells in the tumor was calculated using a custom-written MATLAB script (21). A cluster was defined as a group of pS6^+^ cells with a distance < 10 μm from each other. Clusters with 5 or fewer cells were excluded from the analysis. Clusters were characterized by calculating: the nearest distance (the shortest distance between each pS6^+^ cell), number of pS6^+^ cells < 100 μm radius (the number of pS6^+^ cells within a 100 μm radius from each pS6^+^ cell), NNI (the ratio of the average nearest-neighbor distance to the simulated average nearest-neighbor distance), cluster density (the total number of clusters per sample volume), clustered pS6^+^ cell density (the total number of pS6^+^ cells within clusters per sample volume), mean number of pS6^+^ cells per cluster (the mean number of pS6^+^ cells per cluster), and clustered pS6^+^ cell ratio (the ratio of the total number of pS6^+^ cells within clusters to the total number of pS6^+^ cells within the whole sample). The number of pS6^+^ cells < 100 μm radius was calculated using the MATLAB function “findNeighborsInRadius,” and the NNI was calculated with our custom-written MATLAB script as previously described (21).

### Tumor vasculature analysis.

The tumor vasculature analysis was performed by preprocessing images of CD34 immunolabeled UTUC samples with ImageJ (“Unsharp Mask” and “Normalize Local Contrast” function if needed) and Amira (“Gaussian Filter” and “Background Detection Correction” function). The preprocessed images were downsized to the isotropic resolution of 4 μm, and the blood vessels were segmented according to the intensity level of CD34 using the Amira software. Then, the vessel attributes were assessed by first removing the image noise (“Remove Islands” function in Amira) and calculating the vessel volume (“Volume Fraction” function in Amira), radius, length, and tortuosity (“Auto Skelton” function in Amira). Finally, the CD34 density (%) was calculated as the ratio of the CD34^+^ volume to the total volume.

### Cell-to-vessel analysis.

The distance between each voxel of the segmented vessel and the closest cell was determined using MATLAB’s “pdist2” function to analyze cells’ distribution around the blood vessels. Next, the volume around the vessel was calculated by counting the number of voxels within 10 μm of the segmented vessel by creating a distance map using the “bwdist” function in MATLAB. Finally, the density of pS6^+^ cells around the vessel was calculated by counting the number of pS6^+^ cells per unit volume surrounding the vessel.

### Statistics.

All quantitative data were collected from experiments performed in triplicate and expressed as the mean ± SD. The statistical tests and definition *n* for each analysis are listed in the figure legends. In the text, *P* values refer to Mann-Whitney *U* tests unless stated otherwise. Mann-Whitney *U* tests (independent samples) and Wilcoxon signed-rank tests (dependent samples) were used for comparisons between the 2 groups. For comparison between simulated and experimental data, the *Z*-test was used. Log-rank tests were used for comparisons between Kaplan-Meier curves, with corrections for multiple comparisons. In the Kaplan-Meier curves, continuous variables were converted into categorical variables by division with the median. When comparing the results of 2D and 3D image histopathology, 2D images were selected from 3D *Z*-stacks by generating uniform random numbers using Mersenne Twister (59). ROC curve analysis was used to assess the ability of 3D DIPCO imaging and 2D imaging to distinguish between low- and high-grade tumors in our UTUC cohort (11). The AUC with a 95% CI for each ROC curve was presented as a quantitative performance score. An AUC value of 1.0 stands for perfect discrimination, and a value of 0.5 stands for no discrimination. The DeLong test was used to compare ROC AUC values. No statistical methods were used to determine the sample size. Statistical tests were performed using SPSS (version 24.0; IBM) and MedCalc (MedCalc Software). Differences were considered significant at *P* < 0.05. The experiments were not randomized, and the investigators were not blinded to the allocation during the experiments and outcome assessment.

### Study approval.

The study was performed according to the Declaration of Helsinki and was approved by the Regional Ethical Review Board in Stockholm (registration no. 2018/849-32). Informed consent was obtained from all patients.

### Data availability.

The raw data used to make the graphs found in the manuscript’s figures are available in the accompanying Supporting Data Values file.

## Author contributions

KF, SK, MB, and PU designed the study. KF and YL performed the experiments. KF, SK, and KT performed 3D image processing. KF and IAR analyzed the data. MB and TAA provided human tumor samples. GJ and SM performed clinical histopathology. MB, ZW, NN, NT, MO, and AM provided conceptual advice. KF, ZW, and PU wrote the original manuscript.

## Supplementary Material

Supplemental data

Supplemental video 1

Supporting data values

## Figures and Tables

**Table 1:**
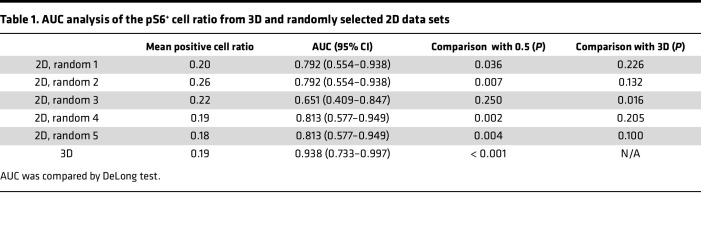
AUC analysis of the pS6^+^ cell ratio from 3D and randomly selected 2D data sets

## Version 1 07/22/2024

Electronic publication
