# Supplementary material for: Three-dimensional imaging of upper tract urothelial carcinoma improves diagnostic yield and accuracy
Source: JCI Insight. 2024 Jul 22;9(14):e175751. doi: 10.1172/jci.insight.175751 (PMC11383588; doi:10.1172/jci.insight.175751)
Supplement: Supplemental data [file jciinsight-9-175751-s110.pdf]

Supplementary Materials for

Three-Dimensional Imaging of Upper Tract Urothelial Carcinoma Improve Diagnostic Yield and Accuracy

Authors: Keishiro Fukumoto[1,2], Shigeaki Kanatani[1], Georg Jaremko[3], Zoe West[1], Yue Li[1], Kimiharu Takamatsu[1], Ibrahim Al Rayyes[1], Shuji Mikami[4], Naoya Niwa[1], Tomas Andri Axelsson[5], Nobuyuki Tanaka[2], Mototsugu Oya[2], Ayako Miyakawa[1,6], Marianne Brehmer[5,7], and Per Uhlén[1]

Affiliations: [1]Department of Medical Biochemistry and Biophysics, Karolinska Institutet, Stockholm, Sweden; [2]Department of Urology, Keio University School of Medicine, Tokyo, Japan; [3]Department of Oncology-Pathology, Karolinska Institutet, Stockholm, Sweden; [4]Department of Diagnostic Pathology, National Hospital Organization Saitama Hospital, Saitama, Japan; [5]Division of Urology, Department of Clinical Sciences, Danderyd Hospital, Karolinska Institutet, Stockholm, Sweden; [6]Division of Urology, Department of Molecular Medicine and Surgery, Karolinska University Hospital, Stockholm, Sweden; [7]Department of Urology and Department of Clinical Science and Education, Stockholm South General Hospital, Sweden.

Table of contents

SUPPLEMENTARY FIGURES 2
Supplementary Figure 1 2
Supplementary Figure 2 3
Supplementary Figure 3 4
SUPPLEMENTARY MOVIE LEGENDS 5
Supplementary Movie 1 5

SUPPLEMENTARY FIGURES

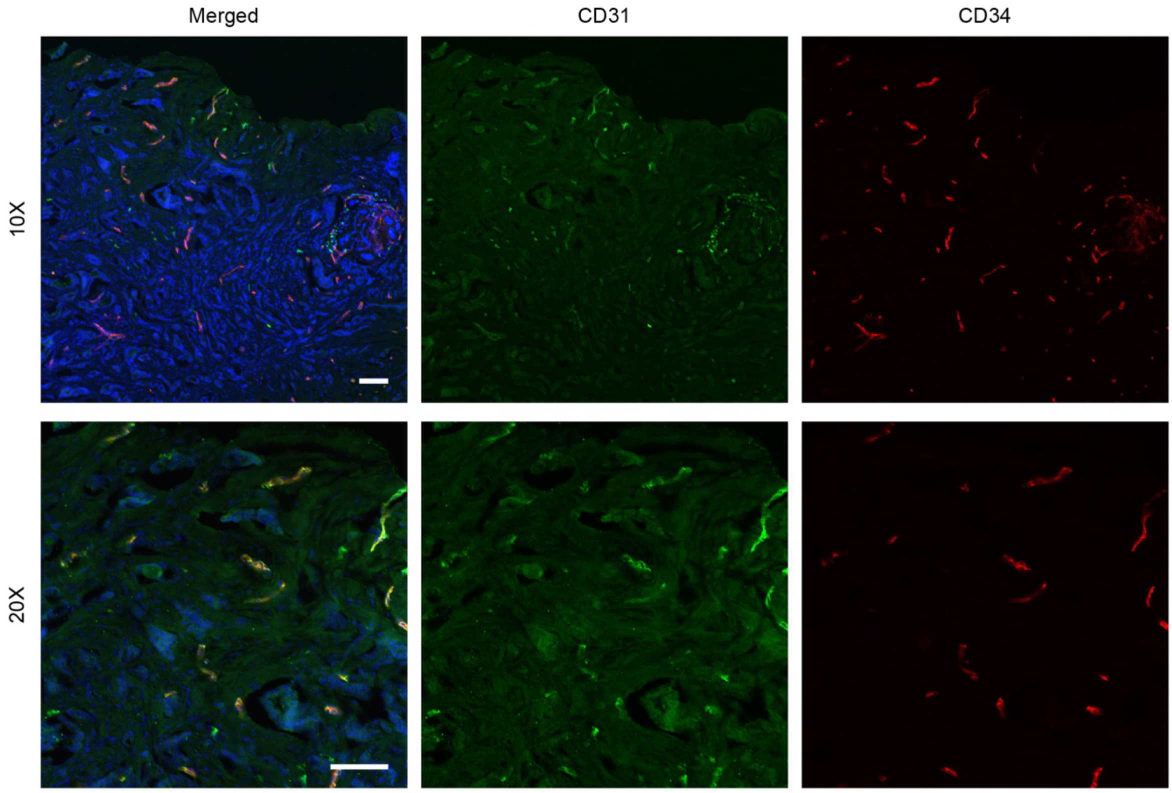

Supplementary Figure 1 | Assessment of blood vessel immunostaining using CD31 and CD34 antibodies. Assessment of blood vessel immunostaining using CD31 and CD34 antibodies at 10X and 20X magnifications. Scale bars, 100 µm.

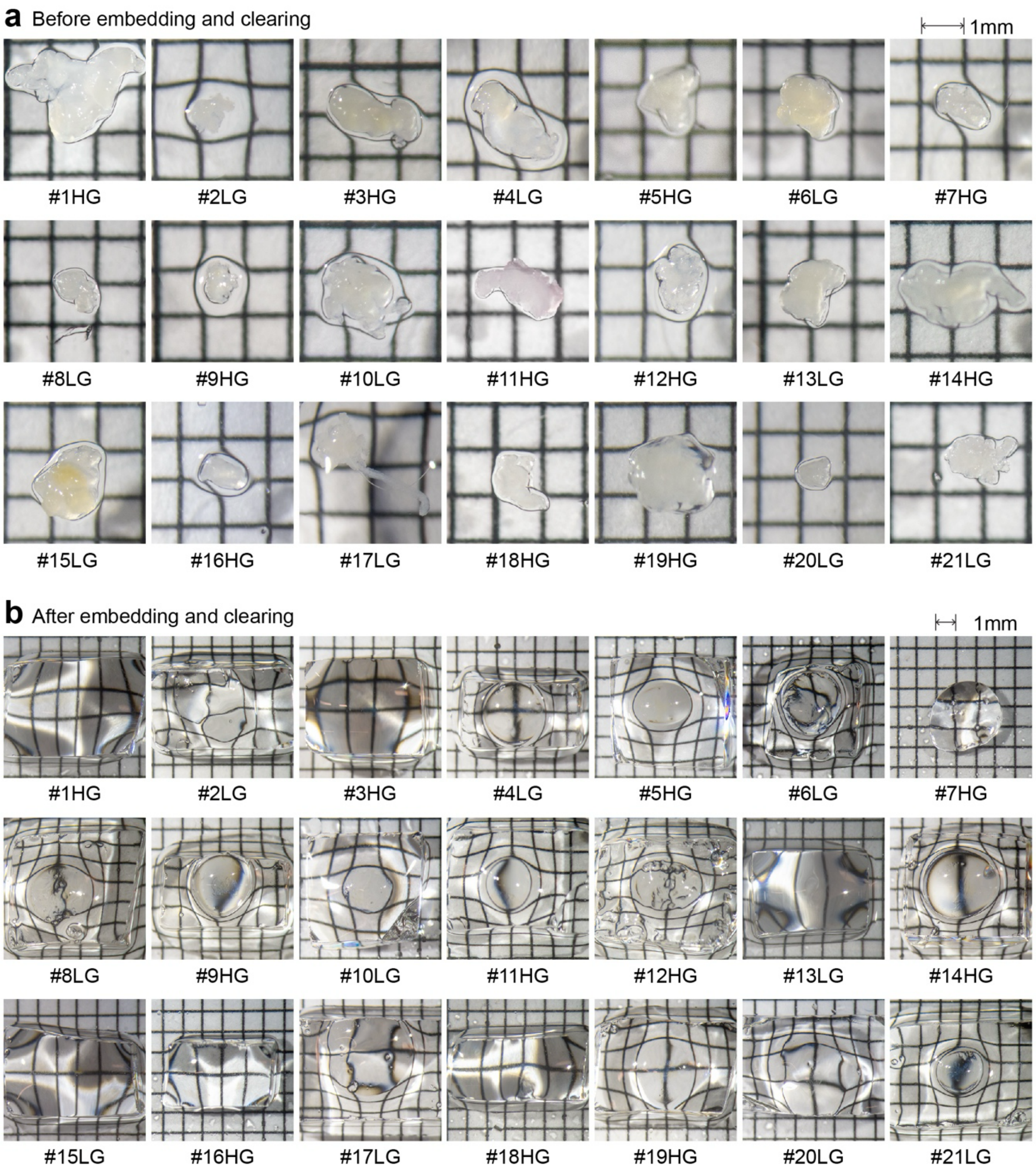

Supplementary Figure 2 | The UTUC cohort. a-b, The 21 UTUC samples used in this study before (**a**) and after (**b**) embedding and clearing. HG, high grade, and LG, low grade. The grid lines are separated by 1 mm.

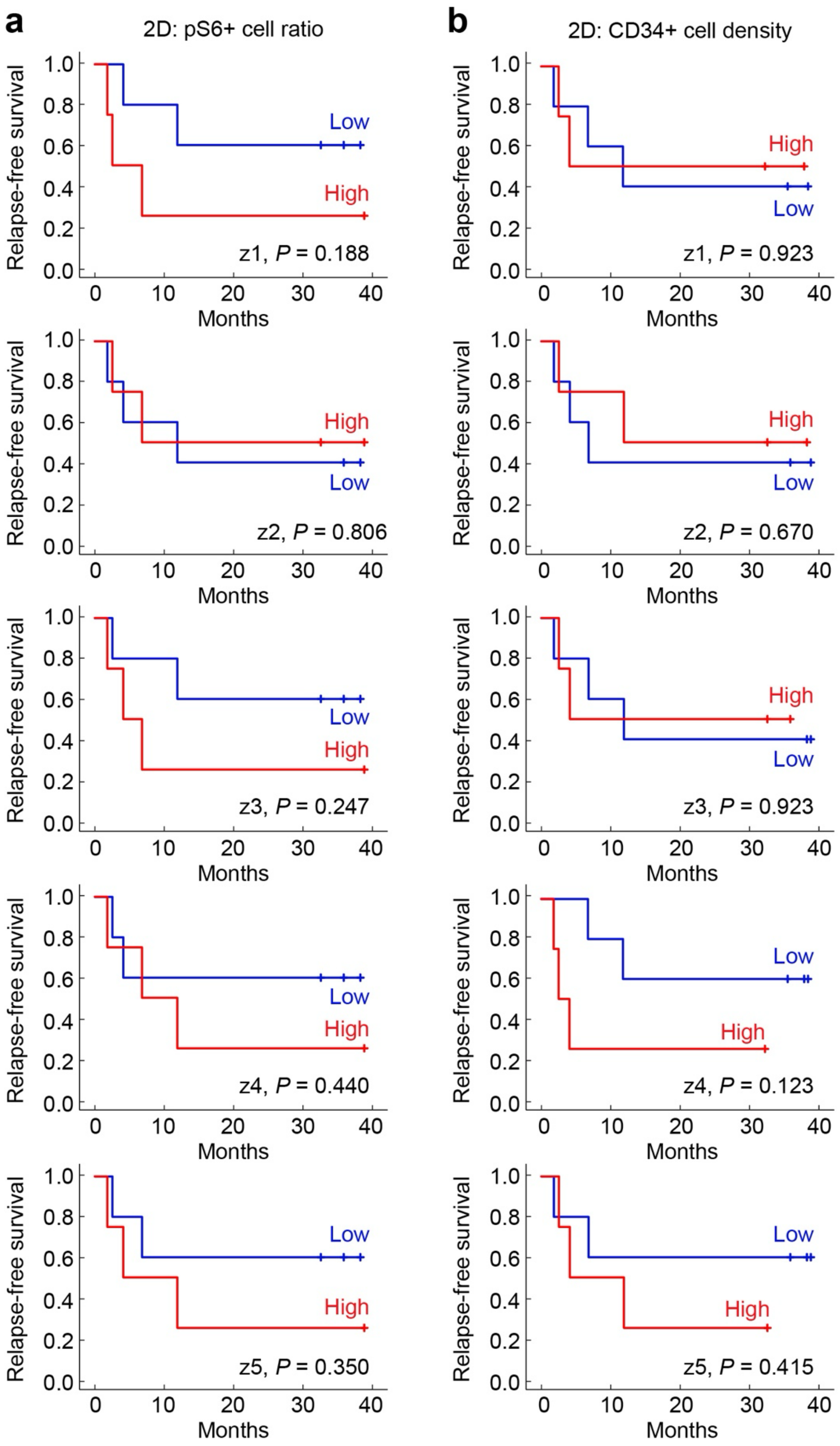

Supplementary Figure 3 | Relapse-free survival analysis in UTUC patients based on 2D imaging. Kaplan–Meier plots of relapse-free survival in patients with UTUC (n = 9) who underwent focal therapy. **a**-**b**, Stratification of UTUC, based on 2D-analysis, into high versus low pS6-positive cell ratio (**a**, z1, P = 0.188; z2, P = 0.806; z3, P = 0.247; z4, P = 0.440; and z5, P = 0.350) and CD34-positive cell density (**b**, z1, P = 0.923; z2, P = 0.670; z3, P = 0.923; z4, P = 0.123; and z5, P = 0.415) in five randomly selected 2D datasets (z1-z5). Statistical analysis was performed using log–rank tests.

SUPPLEMENTARY MOVIE LEGENDS

Supplementary Movie 1 | Volume rendering of pS6 and the vasculature in UTUC. Three-dimensional volume rendering of pS6 and the vasculature in low-grade UTUC sample #10LG. The sample was immunostained for pS6 (red) and CD34, with pseudo-color blue indicating thin vessels and red indicating thick vessels. Nuclei were stained with histone. Bounding box, 1,879 × 1,447 × 920 µm.
File: UTUC3D-SuppMovie-S1.mp4, 24.8 MB.
